# What constitutes problematic khat use? An exploratory mixed methods study in Ethiopia

**DOI:** 10.1186/s13011-017-0100-y

**Published:** 2017-03-21

**Authors:** Awoke Mihretu, Solomon Teferra, Abebaw Fekadu

**Affiliations:** 1 Department of Clinical Psychology, Amanuel Mental Specialized Hospital, Addis Ababa, Ethiopia; 2 Department of Psychiatry, School of Medicine, College of Health Sciences, Addis Ababa University, Addis Ababa, Ethiopia; 3 Department of Psychological Medicine, Centre for Affective Disorders, King’s College London, London, UK

**Keywords:** Problematic khat use, Exploratory mixed methods, Ethiopia

## Abstract

**Background:**

Khat is a psycho-stimulant herb, which has been in use in traditional societies in East Africa and the Middle East over many centuries. Although khat is reported to cause various health problems, what constitutes problematic khat use has never been systematically investigated. This study explored the acceptable and problematic uses of khat from the perspective of users.

**Methods:**

The study used a mixed methods design (exploratory sequential) in which qualitative (emic) data were collected to develop a framework to define problematic khat use. The qualitative data were gathered through in-depth interviews (*N* = 13) and focus group discussions (*N* = 34). By supplementing the emic experiences considered to constitute problematic khat use with an etic definition, DSM-5 criteria for stimulant related disorders, a structured questionnaire was developed. Subsequently a cross-sectional evaluation of 102 respondents was carried out. Respondents both for qualitative and quantitative study were selected through purposive sampling and snowballing methods. Qualitative data were transcribed and subjected to thematic analysis whereas quantitative data were analyzed using descriptive and nonparametric statistics.

**Results:**

Khat use was acceptable socio-culturally and for functional purposes. However, even in these acceptable contexts there was a restriction to the amount, frequency and type of khat used, and in relation to the experience of the individual using khat and other personal characteristics. More specifically, khat use was considered problematic if there was: 1) Impairment (in social and occupational functioning); 2) loss of control in the use of khat; and 3) withdrawal symptoms when not using khat. Among the participants who use khat (*n* = 102), 45.1% (*n* = 46) used khat on a daily basis. The commonest indicators of problematic khat use endorsed by the khat users were loss of control over chewing (73.5%), continuing use of khat despite harm (72.5%) and efforts to avoid withdrawal from khat (61.8%).

**Conclusion:**

Despite reported religious, sociocultural and functional benefits to the use of khat, those with defined problematic khat use have impaired mental health, and social and occupational performance. Comparison of these respondent defined indicators of problem behavior matched almost completely to the DSM-5 (etic-defined) understanding of problematic stimulant use. Although the findings have relevant clinical, research and policy implications, the study focused on users purposively identified. Future larger scale definitive studies are required to make concrete policy recommendations.

## Background

Khat refers to the psychoactive leaves and shoots of the shrub *Catha edulis* [1]*.* Fresh leaves of khat contain the amphetamine like stimulants cathinone and cathine which are extracted by the action of enzymes in saliva. Khat cultivation is more common in Eastern Africa and the Arabian Peninsula [2, 3]. Khat is believed to be native to Ethiopia and was introduced to Yemen between the first and sixth centuries (AD). The chewing of Khat was recorded in the chronicles of the Ethiopian King, Amda Seyon (1314–1344) [4].

Catha edulis has various local names: khat, *qat*, *chat* or *miraa*, ‘*tea of the Arabs’* or ‘*Abyssinian Tea’* in which the dried leaves of khat were boiled and used as modern tea [5].

Cathinone, found in the fresh leaves of khat, and cathine, are the main active ingredients of khat although over 40 compounds have been identified in khat extract [6, 7]. The pharmacological effects of khat chewing were analogous to those of amphetamine and cathine and cathinone were assessed as meeting the criteria for control under the convention of psychotropic substances and recommended for scheduling [8].

The reported psychiatric adverse effects of khat use were: depression, insomnia, suicidal ideation, feeling of anxiousness and irritability, insomnia, loss of appetite, nausea and vomiting, difficulty of seeing at night, headache, fast heart rate, difficulty with balance and coordination, blurred vision, difficulty in concentration, numbness and central nervous system deficits than nondependent chewers [9–12]. Over signs and symptoms there are also psychiatric disorders which were linked to khat chewing [13, 14], including personality disorders, short-lived schizophrenia like psychotic illness, mania and more rarely depression [15, 16]. However, lack of good data to support the link between khat use and any of the major psychiatric disorders has been highlighted [17].

Regarding the physical adverse effects of khat, the WHO Expert Committee on Drug Dependence and others had reported cardiovascular (tachycardia, palpitations, hypertension, arrhythmias, vasoconstriction, myocardial infarction, cerebral hemorrhage and pulmonary edema), respiratory (tachypnoea and bronchitis) and gastrointestinal system (dry mouth, polydipsia, dental caries, periodontal disease, chronic gastritis, constipation, hemorrhoids, weight loss, duodenal ulcer, upper gastro-intestinal malignancy) adverse effects [6, 18].

In addition to its negative health impacts, khat use has also adverse social consequences. Evidence relating to links between khat and loss of relationships has been generated largely through qualitative studies. For instance, loss of relationships with children and weakening of family relations even when the use was considered socially acceptable has been reported [19]. Daily users of khat also consume large fraction of the family budget and khat is purchased at the expense of other important family items such as meat and fruit [20]. The study found out nearly 16% of the family income is said to be spent on khat and many families do worry about the amount of money spent on khat.

Particularly in Ethiopian context, khat chewing was practiced by older men in areas where Islam religion was dominant (Eastern, South and South Western part of Ethiopia). During this time, khat use had been embedded to culture and practiced as a social custom and like other countries(Kenya, Somalia and Yemen) [21]. The pattern of use was also for concentration during functional purposes and praying [22]. Through time, the demand increases along with khat cultivation in different areas of the country and it becomes practiced by youth and females as well. In the present times, different groups of people use khat to increase performance at work including academic tasks, to avoid unpleasant feeling, peer pressure, socialization, and when they have petty time [22–25].

The current prevalence of khat use varies from region to region as determined by its cultivation. Accordingly, in the Northern part of Ethiopia, Tigray regional state, where cultivation is not common, the prevalence of use is the lowest (1.1%) and the highest (53.2%) was reported from Harari, Eastern part of Ethiopia [22]. Among high school and college students the prevalence extends to 64.9% [26]. In addition to Muslim religion and male sex, khat use is also associated with alcohol drinking and cigarette smoking [22, 24, 25, 27]. Cigarette was mainly abused to maximize the stimulation power of khat (during chewing) and alcohol is practiced to break the aftereffect of khat.

Generally, some of the studies focus on the potential negatives of khat use and others emphasize the benefits of khat. What exactly constitutes problem khat use is poorly defined and poorly investigated. Although there are several studies looking at the potential negative consequences of khat use, what constitutes harmful khat has never been explored meaningfully. Most reports focus on prevalence of khat use without defining what the prevalence represented. Additionally, more focused proposals and explorations of targeted interventions (and preventions) could not be carried out if we do not have established methods to identify people who are problematic users. The main aim of the current report is to define what constitutes problematic khat use among khat users in Addis Ababa, Ethiopia.

## Methods

### Study design

The research employed a mixed method, exploratory sequential design. In the initial qualitative phase of the study, we carried out an in-depth exploration of the behavior of khat use and what constituted acceptable and problematic khat use. Based on this exploration it was possible to establish an emic construct of problematic khat use. In the quantitative part of the study, signs and symptoms considered to be indicators of problematic khat use during the initial phase were combined with signs and symptoms of stimulant use according to the international (“Western”) definition of the DSM-5 to assess the pattern of problematic khat use among khat chewers.

The qualitative method relied on a phenomenological approach with limited ethnographic exploration. Both in-depth interviews and focus group discussions were employed. The quantitative part of the study relied on cross-sectional assessment of users.

### Participants and sampling

Eleven khat users and two family members of khat users were selected for the in-depth interview. Four focus group discussions (FGD), with nine khat users in each group, were also conducted. One FGD was composed of female respondents and 102 participants participated for the quantitative part of the study. The participants for both qualitative and quantitative parts of the study were recruited purposively. They were recruited from khat markets and khat cafes. Finding female khat users was very difficult because it is not customarily for a woman to buy khat publically and chew in khat cafes. Therefore, we make snowballing. Through the process of data collection those participants with better knowledge about khat use behavior were participated in the FGD. The study was conducted in Addis Ababa, Ethiopia in 2014.

### Data collection methods

In-depth interviews and FGD were conducted by using semi-structured interview questions and topic guides. The qualitative study identified the key indicators of problematic khat use. These indicators were compiled into a checklist. This checklist was supplemented by items from the DSM-5 indicators of stimulant related-disorders if there were any additional symptoms or behaviors that were not mentioned by the participants during the qualitative study. The checklist (questionnaire) which was developed through the initial qualitative study was surprisingly similar to the DSM-5 indicators of stimulant related disorders and only few additional items were included from the DSM-5. Kessler Psychological Distress Scale (K-10), Oslo social support scale (OSS-3), Fast Alcohol Screening Test (FAST) and List of threatening experiences (LTE) were used to assess psychological distress, social support, harmful drinking and threatening experiences respectively.

### Data analysis

Audio taped in-depth interviews and focus group discussions were transcribed in to Amharic, the official language of Ethiopia. Data were coded and thematically analyzed. The quantitative data were analyzed using simple descriptive summaries: frequency, percentage and mean of various factors and outcomes. A non-parametric test called Mann-Whitney *U* test was used to analysis the difference in psychological distress, social support, harmful drinking and threatening experiences between high risk and low risk groups of problematic khat users. Mann-Whitney *U* test was selected because the sampling technique was not probability and normality was not assured. The statistical Package for Social Sciences, (SPSS version 20) was used in analyzing the data.

### Ethical consideration

The study was approved by the Ethics committee of the School of Psychology, Addis Ababa University. A support letter was also written by the School of Psychology. All personal identifying information was not processed for data analysis. Data collection was conducted confidentially after obtaining informed consent, which also included consent for the digital recording of interviews for those who participated in the qualitative interviews.

## Results

### Acceptable khat use

#### Qualitative results

Seven women and 27 men participated in the FGD and 13 participants were also participated in the in-depth interview. The majority of the participants’ age was between 32 and 40 years (Table 1). Most (*n* = 26) had at least high school diploma. Only four were unemployed.

**Table 1:** Socio demographic characteristics of participants for qualitative study (*N* = 47)

	In- depth interview khat users	In- depth interview Family members	FGD
Number of respondents	11	2	34
Age			
20–30	2	-	3
32–40	5	1	26
41 and older	4	1	5
Gender			
Male	11	1	27
Female	-	1	7
Marital status			
Unmarried	4	-	7
Married	5	2	22
Others^a^	1	-	5
Schooling			
Elementary school and below	3	-	8
High school-diploma	4	-	20
Diploma and above	4	2	6
Religion			
Muslim	5	1	19
Orthodox	4	1	12
Protestant	2	-	3
Employment			
Employed	6	2	17
Private business	4	-	8
Jobless	1	-	4
Others^b^	-	-	5

^a^ divorced, separated due to death ^b^students, daily laborers

Respondents associate the acceptable practices of khat chewing with sociocultural, functional and medical benefits.

### Functional and socio-cultural acceptability of khat use

Although all the respondents did not agree that there is acceptable khat use, all agreed that khat has been useful for improving performance in specific tasks, comforting mourners and to making joyous celebration in weddings. Khat was assumed to be the most important agent for socialization and social group formation. It was argued that in some societies where khat uses is more common, like Harar in the Eastern part of Ethiopia, people chew in social groupings and they share different life concerns during chewing sessions. Respondents have also used khat to alleviate pain. Female discussants reported the practice of drinking *aweza* (hot beverage made from boiling dry or fresh khat leaves in water) to induce abortion. The following quote is a summary of the acceptable use of khat.

*In my opinion, if khat is chewed for a specific purpose, I do not think it is a problem. If I chew khat for praying, it is not a problematic khat use and it is also normal using for social life: wedding, condolence, idir/afosha and other social gatherings*. (FGD#3, age 33)

And when another respondent reported the socialization issue;

*People in Harar (East Ethiopia) usually chew khat at verandah with groups since the weather is hot. The groups of people that are gathered for chewing are called jema (individuals gathered to gether for khat session) and afosha (muslim idir). The khat session starts 12:00 am and ends as members’ interest and they chew khat turn by turn at members’ house.* ((FGD #2, age 28)

### Unacceptable use of khat

Respondents advised to limit the amount of khat used, the frequency of use and the type of khat, depending on the experience of person using khat and other personal characteristics. Concerning the frequency, khat should be chewed less frequently and in variable intervals. The respondents explained that for better functionality, the amount should be managed and they condemned adventurous chewers who chew more than a bundle just merely to show off or inflate them or to pretend to be rich. Respondents also admonished that khat should be chewed in the afternoon. Morning sessions are not advised and not acceptable. Respondents including women users themselves said that it is not acceptable for women and children to chew khat. Although women and children chew khat, it was not frequently observed when they buy khat or holding khat publically in Addis Ababa. Men respondents worried about the rapid increase in the habit of khat chewing among women and high school students. It was not recommended and not acceptable for men and women to chew khat together because from their experience when both sex chew khat together, they tend to engage in unplanned and risky sexual intercourse especially among the young.

### Problematic use of khat

Problematic khat use was described in terms of quantity of khat used, amount of time spent in using khat, the immediate and longer term effects of use, and in terms of effects of cessation of use.

### Quantity of khat used

Through time, to get the stimulating effect of khat, users either increase the amount of khat they chew or use additional stimulants like caffeine and nicotine. Another indicator of taking an increased amount of khat was chewing the *geraba* (leftover khat) and poor quality khat. Almost all of the respondents agreed that *“if we have money, we always want increased amount and quality khat.*” Too much amount of khat is best understood in terms of increasing the amount of khat through time.

### Time spent on the behavior

Respondents blame khat for the amount of time needed to engage in the chewing behavior and for the behaviors, which occur following the chewing sessions. Here, what one respondent said is instructive. *“Khat kills your time while chewing, but waiting for the session of chewing also kills you.”*(IDI#7, age 35). Another respondent also confirmed this by saying:

*As far as my experience is concerned, if someone chews in scheduled time, that will be no problem. But as of me there are individuals who are not khat addicted but have time addiction problem. I am impaired in all activities unless I get khat in my regular time (1:00 pm). (IDI#11, age 24)*

The most problematic users were those who chew the whole day starting from morning, which they term as ‘*yejebena*’, through noon – ‘*ayre’--* and in the night – ‘*katira”.* The purpose of chewing in the morning is to open their eyes and to stay alert. Those who chew the whole day or night are ignoring other important duties of life.

### Feeling high (M*irqanna*)

Feeling high (*mirqanna*) was defined by the respondents as distressing overstimulation, which is beyond the control of the user. During *mirqanna* state, there are different signs and symptoms that cause significant subjective distress or impairment in occupational, social and other important areas of functioning even if they want it to be euphoric. The reported symptoms of *mirqanna* were dilated pupil, feeling uneasy, internal fear, involuntary movements of lips, hands, tongue or mouth, which the individual can’t control, and feeling restless and taking long walks without apparent purpose. Increase in goal directed activities such as extravagancy, urgency for sex and drinking alcohol, doing or planning unachievable tasks. Some chewers may be mute while others become talkative with flight of ideas. They get easily annoyed, fearful or cheerful. Cognitively, what is expected and acceptable is improved attention and concentration but respondents also reported experiencing exaggerated attention and recall of information which may lead to confusion. Physiologically; increased body temperature and pulse rate were experiences which occurred during and immediately after chewing khat.

Other complaints of chewers were perceptual disturbances. For instance, they feel easily frightened by insignificant external stimulus, especially sound and touch. Another was being hyper-vigilant. Others reported perceiving a sound to be too near; when in reality it is very far. Confusion and poor recognition of familiar environment, which occasionally resulted in car accidents was also reported by khat users as a consequence of the *mirqanna*. Moreover, misinterpretation of external stimulus was also reported. Here are statements from the respondents:

*In a taxi or a bar I am frightened to express what I want because I believe that they know that I chewed khat and they might think that I did something wrong or committed a crime. When people talk to each other, I take it as if they are talking about me. I also excessively fear when my phone rings; I can’t talk. I hear the voice in the left side when someone is in fact speaking to me from the right side. (FGD# 2, age 36)*

*I feel disturbed when socks fall from the rope. The socks were hanged from outside home while I am in my bed.* (FGD#2, age 34*)*

*Whenever I chew, I will be tormented by insects/louse in my bed during sleeping, but this does not happen if I didn’t chew. My wife also confirmed that the bed was clean and free of insects*. (IDI# 10, age 43)

The above behavioral effects depend on the individual and the type of khat. These behavioral and physiological changes are managed normally through *chebsi* (reversing the after effects of *Mirqanna* through various activities). Most respondents drink alcohol for this purpose and others perform risky sexual intercourse (with commercial sex workers or any other person). Religious chewers, especially Muslims, reverse the aftereffects by drinking milk. Those who haven’t money to reverse the *mirqanna* through *chebsi* were the most affected by the unwanted signs and symptoms.

### Withdrawal experiences of khat use

Users of khat reported different withdrawal signs and symptoms of khat. These signs and symptoms occurred when users stop their use and/ or when they reduced the amount of khat they use. Most of the time, the users’ experience withdrawal symptoms close to the time of their regular time of use (usually *ayeria* time). The reported withdrawal experiences were increased appetite, increased sleep, yawning, decreased energy, irritability, loss of motivation and concentration, restlessness, craving, depressed mood and unpleasant dreams. In order to manage these withdrawals some respondents chew khat in the morning, which is named *yejebena*. Others who didn’t chew in the morning, did not chew in order to save their money for the afternoon khat session and not because they did not experience withdrawals. These users try to manage the withdrawal symptoms in the morning by taking excess caffeine and smoking cigarette.

Here are two respondents experience of withdrawals:

*I stopped chewing khat for 4 months due to work (training), and it was also difficult to get khat. From the first day of abstinence, I was irritable, easily fatigued and sleepy and I couldn’t attend the training. I frequently missed the lectures. (FGD#1, age 29)*

*The stupid part of khat is the unpleasant dreams (* ***dukake*** *) which are experienced when you didn’t chew and when you were at* ***Wusewase*** *level –reduced the amount. You see horrific dreams; seeing while snake, hyena and other dangerous animals come to you. One day I bought socks with my khat budget, and then I went to bed without chewing. During the night I was tormented a lot by unpleasant dreams. A strange man was punishing me saying I gave you the money for khat; why did you buy socks? He forced me to chew the socks. I [actually] chewed the socks during my dream. (FGD#2, age 35)*

The behavioral effect, which is experienced by users as a result of decrease in the amount of khat is called ***wesewase*** (failure to achieve the highs of khat). The term, *wesewase* is also used to describe the state of craving when one sees some cues of khat use, such as seeing khat leaves. One respondent said: *“You can’t always get money for khat regularly. By this time you are forced to divide up the existing amount of khat or if you are chewing being in Jema, what you have to do is mefalate (*  *), which means chewing speedily. But in all circumstances you will be at wesewase. This is typically observed when guests join the khat session without having their own share.”* (FGD #2, age 29)

### Desire to stop or cut down without success

Respondents expressed persistent desire to stop or cut down khat chewing. The respondents cited wasting of time, psychological dependence, pressure from family or friends, the catalyst nature of khat (its tendency to push one to do other things), overall health impact, psychosocial and economic harms as major reasons for the desire to stop.

One respondent said that;

*Not only the khat pushes you to stop but the associated drugs and activities also force you to stop khat. khat is akatari (catalyst). If you didn’t chew you will not feel internal pressure to smoke, drink alcohol or to engage in risky sexual intercourse.* (IDI# 5, age 35)

However, some users find it difficult to stop as illustrated by the following examples.

*It is difficult to totally abstain from khat. I even start to chew again after stopping for one year. By now I am not confident enough to stop and I can’t make an oath as I can, because I know myself. The reason why I stop was the problem with the poor quality of the khat itself. When the khat becomes (of good) quality and when I see my friends’ enjoying it, I started again.* (FGD#2, age 34).

*I have stopped for 4 years. I decided to stop suddenly. Later on the problem is you would decide that you can’t accomplish any serious tasks without khat; otherwise the khat didn’t force you to chew. Thus I start chewing again*. (FGD#2, age 35)

*You stop and then you start to use it again. I have stopped using khat but it fails because of my friends influence. Most of the times you decide to stop at the evening while you suffer loss of sleep. But while you start chewing after stopping, you blame that time you decided to stop.* (IDI#10, age 43)

### Social and occupational impairment

The social or interpersonal problems due to khat were related to the acceptance of family members and the pattern of khat use behavior. The problem is more serious for females because khat chewing is not generally acceptable in the culture. Khat chewers also have limited time to their families and to attend social gatherings and/or festivals.

*My friends insist on me to stop khat because I didn’t give them time. I have also a deprived social life. I don’t attend weddings, funerals, Idir or other social gatherings. Due to these behaviors no one gives me any social responsibility. (*IDI#5, age 35)

### Hazardous use

There were multiple reports of khat use in physically hazardous situations. These are mostly during *mirqana* or *harara (*craving*).* The risks mostly occurred while driving a car, working at a machine and electricity. Respondents relate the occurrence of risks to increased mental and physical tempo, underestimating danger (over confidence). In other cases, their mind would be preoccupied by thinking about *chebsi* (alcohol or other things). There were respondents who prefer to persist in khat use despite frequent accidents, specially driving a car. A driver concluded that *“khat is a fuel for the driver, as Benzene is a fuel for the car.”(IDI#6, age 35*). The problem will be worse when alcohol is taken in addition.

### Effect on child (female focus group discussion)

Women reported that they observe different behavioral changes in their baby when they breastfeed their child after chewing khat. The baby sleeps poorly, cries and screams and appears to have abdominal pain. Respondents also asserted that khat use resulted in decreased body weight of their child. They added that even if the child was healthy initially, it will be undernourished subsequently because chewing khat decreases the appetite of mothers, which in turn decreases the breast milk production. These women continue using khat while knowing and observing the the negative effect of their behavior/chewing khat on their baby.

### Quantitative results

#### Demographic characteristics and chewing behavior

Most of the participants were men (*n* = 80; 78.4%), single (*n* = 71; 69.6%), Christians (*n* = 76; 74.5%) and Amhara (*n* = 41; 40.2%) (Table 2). Nearly half of the participants (49.0%) had started chewing khat before the age of 10 (Table 3). Majority of the participants (*n* = 35; 34.3%) chewed khat on a daily basis with regular session of both at in the morning and afternoon from 3:00 pm 3:00 pm (*n* = 36; 35.5%). Participants chewed different types of locally planted khats and quantification of the amount used depend on the type of khat (Table 3). The socio demographic and chewing characteristics of participants are summarized in Tables 2 and 3.

**Table 2:** Demographic characteristics of Background information of participants in the quantitative study (*N* = 102)

Characteristics		Number	Percent
Age	15–24	24	23.5
	25–34	45	44.1
	35 and above	31	30.4
Sex	Male	80	78.4
	Female	22	21.6
Religion	Orthodox	66	64.7
	Muslim	24	23.5
	Protestant	10	9.8
	Other^a^	2	2.0
Marital status	Single	71	69.6
	Married	24	23.5
	Divorced	6	5.9
	Other^a^	1	1.0
Living arrangement	With parents or other	60	58.8
	Relatives		
	With partner	15	14.7
	Alone	26	25.5
	Other^b^	1	1.0
Relative Wealth	Low	38	37.3
	Medium	54	52.9
	High	8	7.8
Ethnicity	Oromo	17	16.7
	Amhara	41	40.2
	Guragie	18	17.6
	Tigria	13	12.7
	Others^d^	8	8.8
Employment	Private business	20	19.6
	Student	12	11.8
	Employed	48	47.1
	Daily laborer	6	5.9
	Jobless	5	4.9
	Petty trade	9	8.8
	Other^c^	2	2.0
Total		102	100

^d^welayta, selti, ^c^Prostitution ^b^quit from home due to khat chewing behavior ^a^window

**Table 3:** General overview of respondents’ khat use behavior

Variable		Number	Percent
Age at start of khat use	Less than 10	50	49.0
	10–19	34	33.3
	20–29	8	7.8
	30 or older	8	7.8
Current average khat use frequency	Less than once a month	6	5.9
	Monthly	4	3.9
	Weekly	18	17.6
	2 to 4 days a week	28	27.4
	Daily	35	34.3
	More than daily	11	10.8
Regular khat session	Morning only Immediately after lunch(after12:00 pm/1:00 pm’)	11	10.8
	Morning and after around 3:00 pm	36	35.3
	Whole day	34	33.3
	Whole night	6	5.9
	Other^a^	9 6	8.9 5.9
Current average khat amount per one session	Quarter bundle	9	8.8
	Half bundle	16	15.7
	One bundle	22	21.6
	Two *wendo*	6	5.9
	More than three *wendo*	5	4.9
	50–100gm^b^	7	6.9
	>100gm^b^	15	14.7
	Unknown amount	20	19.6
Type of khat used regularly	*Gelemso*	12	11.8
	*Wendo*	24	23.5
	*Bahir dar*	17	16.7
	*Guragie*	21	20.6
	As available	18	17.6
	Other^c^	9	8.8
Total		102	100

^a^No defined time^b^ Bahirdar Khat which is quantified by grams ^c^ *colombia, beleche, hidna*

### Problematic khat use

Indicators of problematic khat use were identified (Table 4). All were identified through qualitative exploration and were identical to the DSM-5 criteria except one, recurrent risky sexual engagement after chewing khat. The commonest indicator of problematic khat use was persistent desire to use khat or unsuccessful effort at cutting down, endorsed by 73.5% of the respondents (*n* = 75/102). Continued khat use despite its harmful use and the use of other psycho-stimulants to avoid withdrawal symptoms were endorsed by 72.5 and 61.8% respectively. Other important indicators of problematic khat use were occupational and social impairment endorsed by over half of the respondents and craving. All the indicators of problematic khat use corresponded to the DSM-5 criteria of stimulant use disorder except risky sexual behavior, which resulted either directly from the *mirqana* or as part of the deliberate effort by the khat user to reverse the negative after effects of the *mirqana*. This behavior was endorsed by about a quarter of the respondent

**Table 4:** Criteria for problematic use of khat

Criteria	Number	Percent
Spending too much time using khat	30	29.4
persistent desire or unsuccessful efforts to cut down or control khat chewing	75	73.5
Craving/*Harara*	31	30.4
Continued khat chewing regardless of its harm	74	72.5
Recurrent khat chewing in physically hazardous situation	22	21.6
A markedly diminished effect/*wesewase/*when chewing a decreased mount of khat than the regular amount	43	42.2
Recurrent risky sexual engagement after chewing khat	25	24.5
The use of other stimulant in an increased amount to relieve or avoid withdrawal symptoms	63	61.8
The use of khat to relieve or avoid withdrawal symptoms	22	21.6
Marked occupational impairment	59	57.8
Marked social impairment	57	55.9
Believes that cannot accomplish a serious occupational or social task without chewing khat	39	38.2

### *Mirqanna*

**Fig. 1:**
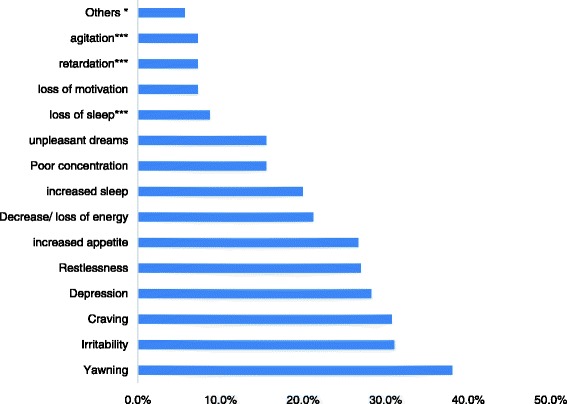
Withdrawal associated experiences of problematic khat use. *** Experiences from DSM-5 criteria not reported from the qualitative but found valid from the quantitative

Physical, cognitive, emotional and behavioral manifestations of *mirqana* were described by the users (Fig. 1). The signs/symptoms of *mirqana* shown in Table 5 were all mentioned in the qualitative exploration except four, which were extracted from the DSM-5 criteria.

**Table 5:** Experiences associated with *mirqanna (*intoxication)

Domain	Experiences	Number	*Percentage*
Thought	Flight of idea	18	17.6
	Unrealistic plans	42	41.2
	Delusion of reference	19	18.6
	Suspiciousness	15	14.7
Perception	Illusion	4	3.9
	Auditory perpetual disturbance	16	15.7
Emotion	Fear	25	24.5
	Irritability	14	13.7
Behaviors	Pressured speech	15	14.7
	Extravagance	37	36.3
	Stereotyped activities	19	18.6
	Communication difficulty	18	17.6
Physical	Pupillary dilation	45	44.1
	Increased sexual desire	21	20.6
	Muscular weakness^a^	19	18.7
	Motoric excitement	17	16.7
	Hyper vigilance	17	16.7
	Hypotension^a^	7	6.9
	Vomiting/nausea^a^	4	3.9
Others	Confusion^a^	15	14.7
	Talking alone	4	3.9

^a^experiences similar to the DSM-5 criteria

### Withdrawal experiences of problematic khat use

Problematic khat users reported different withdrawal signs and symptoms (Fig. 1) that occurred when users stopped their use or reduced the amount they used. Again most of these experiences were reported spontaneously during the qualitative exploration although the experiences match with the DSM-5 list of symptoms for withdrawal in stimulant use disorder.

**Fig. 2:**
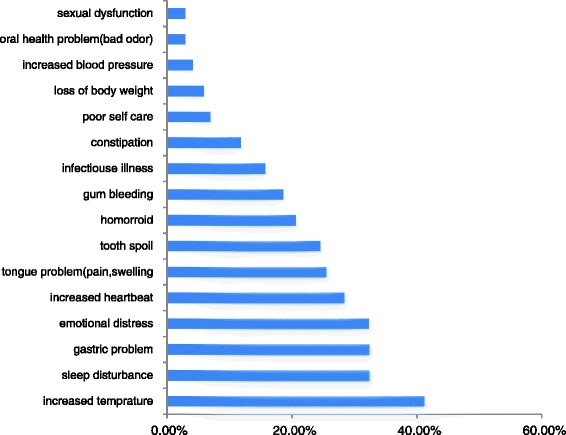
Reported health impacts of problematic khat use

### Problematic khat use and reported health effect

Different physical health impacts of problematic khat use were reported (Fig. 2). Many of the respondents (81.4%) have at least one physical health complaints. The top five complaints were: increased subjective temperature (41.2%), sleep disturbance (32.5%), gastric problem (31%), emotional distress (31%) and increased heart beat (28%).

**Fig. 3:**
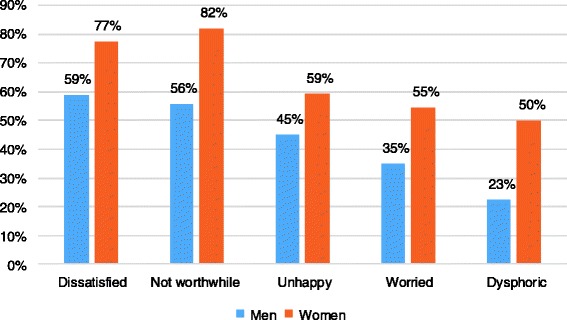
Overall satisfaction domains stratified by sex (Although worse level of satisfaction among women, only dysphoria significantly higher in women; *p* = 0.017)

### Impacts of problematic khat use

The psychological wellbeing, social support, drinking behavior, facing threating experiences and overall satisfaction of khat users were examined. The descriptive finding revealed that about half of the participants (*n* = 55; 54.5%) were problematic alcohol drinkers. Other majorities of the participants experienced serious social problem with friends, neighbor or relatives (*n* = 41; 40.2%), divorce (*n* = 37; 36.2) and financial difficulties (*n* = 35; 34.5%). They also had poorer overall life satisfaction scores although this seems to be worse among women (Fig. 3).

Mann-Whitney *U* test was used to examine the difference in psychological distress, social support, harmful drinking and experience of threatening events between low and high risk problematic khat users. Low risk and high risk is operationalized as below and above the median (the difference between median and mean value was too minimal). Those with higher level of problematic khat use pattern had significantly higher levels of psychological distress (*p* = 0.007) and harmful drinking pattern (*p* = 0.009) (Table 6).

**Table 6:** Mann-Whitney *U* test summary table for impacts of problematic khat use

Variables	Problematic khat use						
	Low risk		High risk				
Psychological distress	Median rank	N	Median rank	N	U- test	Z	*p*
	6	51	10	49	518	-2.71	.007*
Social support	10	51	10	49	1129	-1	.32
Harmful drinking	.00	51	6	49	922.50	-2.61	.009*
Threatening experiences	27	51	26	49	1093	-1.10	.30

*p < 0.005

## Discussion

This study was conducted primarily to determine what constituted problematic khat use. Despite the increasing interest in the harmful effects of khat and the interest to ban the use of khat in some high income countries like the UK [6], little is known about what constituted problematic khat use. Employing emic and etic approaches, this study investigated what constituted problematic use primarily from the perspective of the users.

Participants considered khat use both acceptable and problematic depending on the context and the pattern of use. The acceptable uses included using for religious, socio-cultural and functional purposes. Denouncing khat use without understanding the context could be in itself problematic. Khat use was considered problematic if there was: 1) Impairment (in social and occupational functioning); 2) loss of control in the use of khat; and 3) withdrawal symptoms when not using khat. Specific indicators were shown in Table 7. These are important indicators which are found in the DSM-5 criteria stimulant use disorders. The harm of drugs is mainly evaluated to its physical harms, dependency and social harms and khat has been listed at the bottom line [28].

**Table 7:** Main indicators of problematic khat use identified through the emic approach

(1) **Quantity of khat**: Using large quantities of khat, the use of increasing amounts of khat through time and the need to use other substances to either enhance the stimulating effects of the khat or to reverse the excessive stimulant effect of khat (*mirqana*). (2) **Time of use**: use of khat in the morning (*yejebena)*, taking too much time to use khat-throughout the day or night and using during unscheduled times and if the users’ life is dominated by khat use. (3) **Repeated excessive negative impact** during or after using khat, including negative behavioral effects (e.g., *mirqana*). (4) **Withdrawal effects** when reducing the amount used or cessation of use, and problematic management of these withdrawal effects. (5) **Craving** for khat. (6) **Desire to stop or cut down** use but unable to do so. (7) **Social and occupational impairments** resulting from khat use (e.g., limited time or no time for socializing, poor self-care, and giving up social responsibilities or activities; being overlooked for social responsibilities; inability to carry out responsibilities within family or work; not having time or money for recreational activities). (8) **Using khat even when it is hazardous** (e.g., operating machinery, driving a car). (9) **Major negative impact on finances**. (10) **Physical harms** resulting from khat use or from withdrawals.	

The emic and etic approaches were overlapping. Defined pattern of problematic khat use can be identified. This study focused on known individuals with established khat use behavior and high level of problematic pattern of use. Within the population, problematic khat use may be minimal. Respondents were mildly (*n* = 3; 3.9%), moderately (*n* = 98; 96.1%), and severely (*n* = 1; 1%) problematic using DSM-5 criteria for classification of risk level. The study was analogous to other studies with etic approach only [29–31] with specific criterion of problematic use, 73.5% of the participants had a practice of persistent desire or unsuccessful efforts to cut down or control khat chewing. This had been repeatedly reported but many chewers fail to succeed.

The study also found out different withdrawal symptoms. The most frequent reported were yawning, irritability, craving and depression. Recent studies reported that Psychological dependency was endorsed by 51% participants in Yemen [30] and 52.2% of khat chewers in Saud Arabia [9] as measured by Severity of dependency scale. Another study among Yemeni residents in UK also showed that 31% of khat chewers reported dependency as measured by American Psychiatric Association Diagnostic Statistic Manual (DSM IV, 1994) [29]. In Ethiopia, from the general population, problematic khat use was found out about 20 and 0.6% chew to avoid withdrawal symptoms [24, 32].

Other problems of khat were health harms. About 81.4% of the participants had at least one health compliant. Other studies also confirmed that khat had mild, moderate and severe kinds of health harm depending on the pattern of use [6, 33, 34]. But as far as the qualitative study revealed it is difficult to conclude as khat itself causes the above health harms. Sugar, candy and other similar substances which are taken during chewing khat to decrease the bitter taste, pesticides of the khat plant and the repeated use of tobacco and alcohol during and after khat use might also be associated with reported health harms.

Recurrent risky sexual engagement after chewing khat which was confirmed by 24.5% of the respondents was also one indicator of problematic khat use. Non problematic khat users didn’t engage to risky sexual engagement due to the feeling high (*Mirqanna)* of khat rather problematic khat users do. This may be because of poor in judgment during feeling high, the effect of sexual desire of khat, chewing together with opposite sex in khat cafes and to reduce the feeling high. Other studies also found that khat chewing results in risky sexual engagement and is a risk behavior for the spread of HIV infection [35, 36].

Other dimensions of khat harm were the functionality and economic impacts. Fifty five point 9 and 57.8% of the participants reported that khat chewing behavior affects their social and occupational functioning negatively. Other studies also reported this [19, 20]. The deterioration of such functioning and a decreased ability to attend social gatherings and/or festivals were commonly reported. The average amount of money spent on khat (about 6 USD) is much higher than the amount of money used to define the poverty line. Adding this to the expenses incurred to support the use of other substances like cigarettes, alcohol, shesha, pea nut, sugar/ candy, soft and hot drinks and rent for sitting at rooms for khat sessions, to the cost of khat chewing could be much more substantial.

One major limitation of the current study was the issue of generalizability. The sampling method employed here was non-probability. The study also employed qualitative and cross sectional quantitative research method which didn’t show causal relationship among variables and the impacts of problematic khat use were only the perceived and experiences of the participants.

## Conclusion

In conclusion, the study demonstrates that problematic khat use can be defined and evaluated. Interestingly there was a major overlap between the emic and etic approaches (DSM-5 check lists of stimulant related disorders) although the list of indicators for problematic use was more extensive in the emic approach*.* There were significant problems related to problematic khat use and Problematic use of khat was prevalent among the study participants. There were also significant distressing experiences related to the aftereffects of excess use *(mirqana)* and withdrawal. Thus, it is better if there is an integrated prevention and treatment strategy for problematic khat use. Further large scale population based and controlled studies are also required.

## Abbreviations

DSM: Diagnostic statistical power
FAST: Fast alcohol screening test
FGD: Focus group discussion
IDI: In-depth interview
LTE: List of threatening experiences
NA: Narcotic anonymous
OSS: Oslo social support
SPSS: Statistical package for social science
WHO: World Health Organization

## References

[1] Kalix P (1984). The Pharmacology of Khat. Gen Pharmac.

[2] Krikorian AD (1984). Kat and its use: an historical perspective. J Ethnopharmacol.

[3] Getahun A, Krikorian A (1973). Chat: coffee’s rival from Harar, Ethiopia. I. Botany, cultivation and use. Econ Bot.

[4] Baasher T, Sadoun R (1983). The epidemiology of khat.

[5] Halbach H (1972). Medical aspects of the chewing of khat leaves. Bull World Health Organ.

[6] Thomas S, Williams T. Khat (Catha edulis): A systematic review of evidence and literature pertaining to its harms to UK users and society. Drug Science, Policy and Law. 2013;1:2050324513498332.

[7] Pantelis C, Hindler CG, Taylor JC (1989). Use and abuse of khat (Catha edulis): a review of the distribution, pharmacology, side effects and a description of psychosis attributed to khat chewing. Psychol Med.

[8] Kalix P, Braenden O (1985). Pharmacological aspects of the chewing of khat leaves. Pharmacol Rev.

[9] El-Setouhy M, Alsanosy RM, Alsharqi A, Ismail AA. Khat Dependency and Psychophysical Symptoms among Chewers in Jazan Region, Kingdom of Saudi Arabia. BioMed Res Int. 2016;2016:2642506. 10.1155/2016/2642506 PMC4789047 27022605

[10] Bhui K, Mohamud S, Warfa N, Craig T, Stansfeld S (2003). Cultural adaptation of mental health measures: Improving the quality of clinical practice and research. Br J Psychiatry.

[11] Al‐Motarreb A, Baker K, Broadley KJ (2002). Khat: pharmacological and medical aspects and its social use in Yemen. Phytother Res.

[12] Cox G, Rampes H (2003). Adverse effects of khat: a review. Adv Psychiatr Treat.

[13] Dhadphale M, Omolo O (1988). Psychiatric morbidity among khat chewers. East Afr Med J.

[14] Yousef G, Huq Z, Lambert T (1995). Khat chewing as a cause of psychosis. Br J Hosp Med.

[15] Dependence WECoD, Organization WH. WHO Expert Committee on Drug Dependence: thirty-fourth report: World Health Organization; 2006.

[16] Odenwald M, Neuner F, Schauer M, Elbert T, Catani C, Lingenfelder B, et al. Khat use as risk factor for psychotic disorders: a cross-sectional and case-control study in Somalia. BMC Medicine. 2005;3(1):5. 10.1186/1741-7015-3-5 PMC554104 15707502

[17] Odenwald M (2007). Chronic khat use and psychotic disorders: a review of the literature and future prospects. Sucht.

[18] Nencini P, Ahmed AM (1989). Khat consumption: a pharmacological review. Drug Alcohol Depend.

[19] Assefa M (1983). Socio-economic Aspects of Khat in the Hararghe Administrative Region (Ethiopia).

[20] Griffiths P (1998). Qat use in London: a study of qat use among a sample of Somalis living in London: Home Office.

[21] Manghi RA, Broers B, Khan R, Benguettat D, Khazaal Y, Zullino DF (2009). Khat use: lifestyle or addiction?. J Psychoactive Drugs.

[22] Haile D, Lakew Y. Khat chewing practice and associated factors among adults in Ethiopia: further analysis using the 2011 demographic and health survey. PLoS ONE. 2015;10(6):e0130460. 10.1371/journal.pone.0130460 PMC4474667 26090658

[23] Aklog T, Tsegay Girmay, Tiruneh Gebeyaw. assessment of substance abuse and associated factors among students of Debre Markos poly technic college in Debre Markos town, East Gojjam zone, Amhara regional state, Ethiopia. Jimma-Minnesota international symposium on mental health and substance abuse (JIMIS),. 2014.

[24] Alem A, Kebede D, Kullgren G (1999). The prevalence and socio-demographic correlates of khat chewing in Butajira, Ethiopia. Acta Psychiatr Scand Suppl.

[25] Gelaw Y, Haile-Amlak A. Khat chewing and its socio-demographic correlates among the staff of Jimma University. Ethiop J Health Dev. 2004;18(3):179-84.

[26] FACJT M (1994). Khat chewing among Agaro secondary school students. Ethiop Med J.

[27] Deressa W, Azazh A (2011). Substances use and its predictors among undergraduate medical students of Addis Ababa University in Ethiopia. BMC Public Health.

[28] Nutt D, King LA, Saulsbury W, Blakemore C (2007). Development of a rational scale to assess the harm of drugs of potential misuse. Lancet.

[29] Kassim S, Croucher R, al’Absi M (2013). Khat dependence syndrome: a cross sectional preliminary evaluation amongst UK-resident Yemeni khat chewers. J Ethnopharmacol.

[30] Kassim S, Islam S, Croucher R (2010). Validity and reliability of a Severity of Dependence Scale for khat (SDS-khat). J Ethnopharmacol.

[31] Duresso S, Matthews A, Ferguson S, Bruno R. Is khat use disorder a valid diagnostic entity?. School of Medicine, University of Tasmania, Hobart, Australia. 2015.

[32] Gebrehanna E (2014). Prevalence and Predictors of harmful Khat use among university Students in Ethiopia. Subst Abus.

[33] Patel SL, Murray R, Britain G (2005). Khat use among Somalis in four English cities: Citeseer.

[34] WHO. Assessment of Khat (Catha edulis Forsk) Geneva: WHO..2006.

[35] Abebe D (2005). Khat chewing habit as possible risk behaviour for HIV infection: A case-control study. Ethiopian J Health Dev.

[36] Kebede D, Alem, D., Mitike, G., Enquselassie, F., Berhane, F., Abebe, Y., …Gebremichael, T. Khat and alcohol use and risky sex behaviour among in-school and out-of-school youth in Ethiopia. BioMed Central 2005. 10.1186/1471-2458-5-109 PMC1274331 16225665

